# How TRPC Channels Modulate Hippocampal Function

**DOI:** 10.3390/ijms21113915

**Published:** 2020-05-30

**Authors:** Roberta Gualdani, Philippe Gailly

**Affiliations:** Institute of Neuroscience, Université catholique de Louvain, B-1200 Brussels, Belgium; roberta.gualdani@uclouvain.be

**Keywords:** mGluR, working memory, spatial memory, persistent activity, afterhyperpolarization, LTP, LTD

## Abstract

Transient receptor potential canonical (TRPC) proteins constitute a group of receptor-operated calcium-permeable nonselective cationic membrane channels of the TRP superfamily. They are largely expressed in the hippocampus and are able to modulate neuronal functions. Accordingly, they have been involved in different hippocampal functions such as learning processes and different types of memories, as well as hippocampal dysfunctions such as seizures. This review covers the mechanisms of activation of these channels, how these channels can modulate neuronal excitability, in particular the after-burst hyperpolarization, and in the persistent activity, how they control synaptic plasticity including pre- and postsynaptic processes and how they can interfere with cell survival and neurogenesis.

## 1. Introduction: Hippocampal Circuitry and Memory 

Memory is the reactivation of a trace laid down by earlier experience [1,2,3,4]. Explicit or declarative memory is divided into short-term (working) memory and long-term memory, such as, e.g., episodic (related to events or experiences) or semantic (related to facts and concepts) memories. The hippocampus and related cortices play an important role in storing and retrieving memories as well as in spatial navigation. In contrast, implicit (unconscious) procedural memory essentially relies on brain areas implicated in motor function, such as the cerebellum and the striatum.

The hippocampus contains Ammon’s horns (Cornu Ammonis: CA) and the dentate gyrus (DG) and is intimately associated to the entorhinal cortex (EC). The axons of layer II neurons in the EC project to the DG and to a lesser extent to the CA3 region through the perforant pathway. Granule cells of the DG send mossy fiber projections to the CA3 pyramidal cells, which, in turn, send Schaffer collaterals to CA1 pyramidal cells, which project back to layer V neurons of the EC. This constitutes the classical trisynaptic circuit of the hippocampus (EC–DG–CA3–CA1–EC), initially described by Ramon y Cajal in the early 20^th^ century [5] and supposed to encode memory. In addition, CA1 pyramidal neurons receive a direct input from layer III cells of the EC through the temporoammonic pathway. Moreover, CA3 cells interconnect through recurrent collaterals. Granule cells of the DG also project to the mossy cells in the hilus and hilar interneurons, which send excitatory and inhibitory projections, respectively, back to the granule cells.

Transient receptor potential canonical (TRPC) channels are largely expressed in the hippocampus. These proteins constitute sensors of the environment, being for example able to sense neuromediators or chemoattractants, and they control fluxes of cations—essentially Na^+^ and Ca^2+^—in and out of the cell. They are therefore able to mediate cellular responses to these signals and interact with important hippocampal functions.

## 2. TRPC Structure and Activation

The canonical family of Transient Receptor Potential (TRPC) proteins has seven members (TRPC1 to TRPC7) that form homo- and/or heterotetrameric Ca^2+^-permeable, nonselective cation membrane channels [6,7]. Based on sequence homology, this family can be divided into three subfamilies: the TRPC1, -C4, and -C5 subfamily; the TRPC3, -C6, and -C7 subfamily; and TRPC2, which is a pseudogene in humans [8]. In contrast to other TRPC channels that are able to assemble in homo- and heterotetramers, creating a variety of different channels, the function of TRPC1 as an ion channel is a matter of debate [9]. Indeed, it is not sure that it can form homotetramers and function on its own physiologically but may rather be an important linker and regulator protein in heteromeric TRPC channel tetramers. Several in vitro studies showed that TRPC1 can form channel complexes with TRPC4 and TRPC5 but not with TRPC3 and TRPC6, and that all other TRPCs exclusively assemble into homo- or heterotetramers within their own subfamilies [10,11]. Co-immunoprecipitation experiments performed on synaptosomal preparations confirmed the presence of TRPC1/4/5 and TRPC3/6/7 complexes in rat brain [12,13]. TRPC1 was, however, shown to also heteromultimerize with TRPC3/6 in vitro [14], but in vivo, this association required the presence of both TRPC1 and TRPC4/5 and seemed to occur only in embryonic brain [15]. In murine adult brain, TRPC1, TRPC4, and TRPC5 assemble in heteromultimers with each other, but not with other TRP family members [16]. In limbic structures, TRPC1, TRPC4, and TRPC5 are the most abundant; they are highly expressed in pyramidal cells of CA1-3 and DG regions of the hippocampus and in the amygdala [15,17,18], TRPC1 being expressed on both cell somata and dendrites, whereas TRPC5 is located on cell bodies [19] but also on presynaptic terminals [20]. TRPC1, TRPC4, and TRPC5 are also expressed in the prefrontal cortex and in the entorhinal cortex [21]. TRPC1 and TRPC4 are expressed in the lateral septum [22]. TRPC2 is essentially expressed in the olfactory bulb [23]. TRPC3 can be found in CA1 and CA3 regions [24,25], TRPC6 has been localized in the molecular layer and in interneurons of the DG [17] and TRPC7 in CA3-recurrent collateral synapses and Schaffer collateral-CA1 synapses [26].

Several activation mechanisms have been proposed for TRPC channels, the most frequently described being the activation by G_q/11_-protein-coupled receptors or by receptor tyrosine kinases via phospholipase C (PLC)-induced formation of diacylglycerol (DAG) and inositol 1, 4, 5-trisphosphate (IP_3_) [27,28,29]. They can be directly activated by phosphatidylinositol 3,4,5-trisphosphate (PIP3), by phosphorylation by protein kinase C (PKC), or through the hydrolysis of phosphatidylinositol 4,5-bisphosphate (PIP_2_) and/or concomitant generation of DAG [30]. However, in contrast to TRPC2 and the TRPC3/6/7 subfamily, TRPC1/4/5 is not directly activated by the PLC product DAG. Recently, Storch et al. reported that the dissociation of the scaffolding proteins Na^+^/H^+^ exchanger regulatory factor (NHERF) 1 and 2 from the C terminus of TRPC5 is a prerequisite for DAG sensitivity [31]. Hence, DAG sensitivity is a peculiar hallmark of all TRPC channels. In TRPC1/C4 or TRPC1/C5 complexes, the presence of TRPC1 seems to decrease the inward current through the channel compared to TRPC4 and TRPC5 homotetramers, but it might allow its activation by specific receptors [9,32,33]. Indeed, the group of Insuk So recently showed that heterotetrameric TRPC1/C4 and TRPC1/C5 channels were activated by direct interaction with activated Gα_q_ [34] and subsequently inhibited by the PLCβ-induced PI(4,5)P_2_ depletion, the release of Ca^2+^ from the reticulum, and the activation of PKC [35]. TRPC channels are also involved in store-operated calcium entry (SOCE) in cooperation with Orai1 channel and activated by STIM1, a sensor of Ca^2+^ contents in the endoplasmic reticulum [36,37,38,39,40,41] (reviewed in [42]). In addition, TRPC channels are activated by lipids such as sphingosine-1-P, arachidonic acid, cholesterol, and others [43,44]. TRPC1/4/5 activity depends on cytosolic Ca^2+^ [45,46], TRPC4 and TRPC5 directly interacting with calmodulin [47,48]. In contrast, TRPC1 is not activated by intracellular Ca^2+^ but is inserted into the plasma membrane in response to Ca^2+^ entry via Orai1 channels [49]. TRPC channels interact with many other proteins that are involved, e.g., in focal adhesions, cytoskeletal linking, membrane vesicle trafficking, cell cycle, and plasma membrane expression. In a recent article, a transcriptome analysis in eight tissues from mice lacking all of the TRPC channels showed that focal adhesions, extracellular matrix-receptor interaction, cytokine-cytokine receptor interaction, and manganese ion transport were modified in both forebrain and midbrain [50]. TRPC channels are involved in a number of mechanosensory processes [51,52]; however, recent papers seem to point out that they are more likely activated by mechanosensitive processes than directly mechanosensitive [53,54]. Finally, TRPC activity can also be modulated by receptors coupled to G_i/o_- and G_s_-proteins that inhibit or activate the adenylate cyclase, leading to changes of cyclic adenosine monophosphate (cAMP) concentrations and to a modulation of protein kinase A. In the nervous system, through the coupling to G_q/11_, G_i/o_, or G_s_ proteins, different TRPC isoforms can therefore mediate the response to several neurotransmitters, such as acetycholine, noradrenaline, serotonine, dopamine, or glutamate (reviewed in [55]). 

## 3. TRPC Channels Modulate Memory: Lessons from Knockout Animals

The role of TRPC channels has been investigated in different hippocampus-related forms of memories. Using trace cued and contextual fear conditioning, Y maze and step-down tests, Xing and colleagues first demonstrated that TRPC1 depletion led to spatial memory impairment in mice [56]. Thereafter, using T-maze, radial maze and Morris water maze, *Trpc1^−/−^* as well as *Trpc1/Trpc4/Trpc5*-triple-knockout (*Trpc1/4/5*^−/−^) mice were shown to present deficits in spatial working memory but not in spatial reference memory [16,57]. They also exhibited deficiencies in spatial memory extinction and in relearning tasks [16,58]. In contrast, compared to age-match controls, *Trpc3^−/−^* mice performed better in contextual fear conditioning test [59]. Finally, mice overexpressing TRPC6 in the forebrain showed enhanced performance in spatial learning and memory [60].

These results encouraged mechanistic investigations to understand how TRPC channels regulate hippocampal memory functions e.g., by modulating brain development, neuronal excitability, hippocampal persistent activity, synaptic plasticity or adult neurogenesis. These processes are further detailed hereafter.

## 4. Functions of TRPC in Neurons and Possible Implication in Brain Development

In the developing brain, the formation of synapses is a complex process involving sequential steps, many of which are regulated by Ca^2+^ signaling. They include neurogenesis, formation of neuronal polarity, migration, axonal path finding, establishment of dendritic morphology, and synaptogenesis [61]. The involvement of different TRPC isoforms in these processes has been studied essentially ex vivo, on neurons in culture (see review by [62]). Radial glial cells exert an attractant guidance signal on migrating neuronal cells. TRPC1 and TRPC3 isoforms are required for growth cone turning responses to microscopic gradients of chemotropic molecules and neurotransmitters such as netrin-1 [63,64] or glutamate. TRPC1 promotes basic fibroblast growth factor (bFGF)-induced self-renewal of embryonic rat neural stem cells [65]. TRPC1 and TRPC4 regulate neurite extension in embryonic stem cell-derived neurons [66]. TRPC1/3 channels are also required for leptin-induced spine formation [67]. TRPC3 and -6 seem to mediate the response to brain derived neurotrophic factor (BDNF) such as cerebellar granule neuron survival in response to BDNF [68], BDNF-induced chemoattraction, or BDNF-induced spine formation [69]. TRPC5 is a negative regulator of neurite outgrowth [70]. Finally, studying rat brain development, Zhou and colleagues showed that TRPC6 was mainly localized in excitatory postsynaptic sites and was important in the development of dendritic spines and excitatory synapses in vitro and in vivo, explaining the fact that overexpression of TRPC6 improved spatial memory [60].

TRPC channels are thus largely involved in proliferation and migration, in neurite outgrowth, in spine formation, or in axon guidance, and even if TRPC knockout animals do not exhibit major morphological abnormalities of the brain, it is clear that observations made on constitutive knockouts have to be taken with care as it can reflect brain development impairment and could be advantageously completed with pharmacological investigations or with acute conditional genetic deletions (e.g., as in [58,59]). 

This being said, let us see how TRPCs modulate the cellular functions that support mnemonic functions, i.e., essentially neuronal excitability, synaptic plasticity, neurogenesis, and cell survival.

## 5. Modulation of Neuronal Excitability

### 5.1. Metabotropic Glutamate Receptors (mGluRs) Regulate Excitability

**Figure 1:**
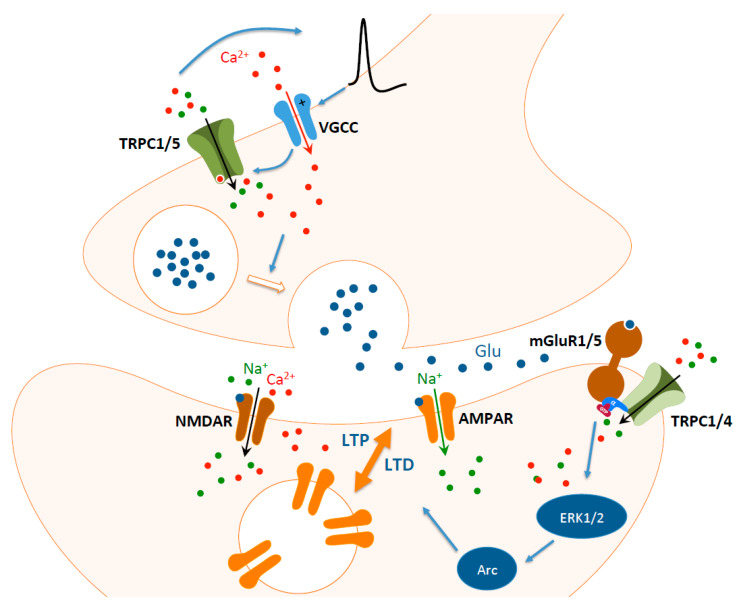
Hypothetical role of transient receptor potential canonical (TRPC) channels in Schaffer collateral-CA1 synapses. In the presynaptic terminal, the entry of Ca^2+^ through voltage-gated Ca^2+^ channels (VGCC) during action potential activates TRPC5 homotetramers or TRPC1/C5 heterotetramers, which increases depolarization and exerts a positive feedback, thereby modulating presynaptic Ca^2+^ dynamics and improving neurotransmission. In the postsynaptic terminal, glutamate released activates α-amino-3-hydroxy-5-methyl-4-isoxazolepropionic acid receptors (AMPAR). If the depolarization is sufficient (for example after a high frequency burst of action potentials), glutamate also activates N-methyl-D-aspartate receptors (NMDAR) allowing an influx of Ca^2+^ that increases the number of AMPAR at the membrane and produces long-term potentiation (LTP). In contrast, low-frequency stimulation increases more modestly postsynaptic [Ca^2+^]_i,_ which decreases the number of AMPAR at the membrane and produces long-term depression (LTD). In both LTP and LTD, a concomitant activation of perisynaptic metabotropic glutamate receptors mGluR1 and/or mGluR5 activates TRPC1/C4 heterotetramers that further depolarize the postsynaptic terminal and induce an entry of Ca^2+^. This, in turn, modulates the activity of extracellular signal-regulated kinases 1/2 (ERK1/2), the subsequent expression of the activity-regulated cytoskeletal-associated protein Arc, an immediate early gene that controls AMPA receptors endocytosis.

mGluRs control neuronal excitability by gating diverse ionic conductances and by modulating synaptic transmission. The group I mGluR comprises mGluR1 and mGluR5 that are coupled to G_q/11_ G-proteins that activate phospholipase Cβ, resulting in the hydrolysis of PIP_2_ into IP_3_ and DAG [71] and specific cationic channels, which enhance excitability by depolarizing neurons [72]. Gee et al. were the first to report that in the hippocampus, the inward current induced by group I mGluR agonists such as (S)-3,5-dihydroxyphenylglycine (DHPG) was mediated by “TRP-like” channels [29]. At the same time, Kim et al. showed that in cerebellar Purkinje cells, TRPC1 could be activated by mGluR1 [73]. The two proteins were localized in perisynaptic regions of dendritic spines and could physically interact. This was however refuted by Hartmann et al., who demonstrated that TRPC3 and not TRPC1 was responsible for slow synaptic potentials and mGluR-mediated inward currents [74]. In hippocampal neurons, we found that DHPG depolarized the cell by about 10 mV by inducing a slow excitatory postsynaptic current (sEPSC) and an influx of Ca^2+^ that were not dependent on stores depletion nor on PLC activation and that were absent in neurons of *Trpc1^−/−^* mice and in neurons in which *Trpc1* gene had been acutely deleted, thus excluding a developmental cause [57] (Figure 1). The current was also inhibited by Pico145, a specific inhibitor of TRPC1/C4/C5 channels. A similar situation seems to occur in the inferior colliculus [75,76], as well as in hippocampal oriens/alveus interneurons where TRPC1 is activated by- and interacts with mGluR1a [77]. 

### 5.2. After Burst Hyperpolarization (AHP)

Some learning tasks produce enduring changes in the intrinsic excitability of neurons by changing the function of voltage-gated ion channels in the axo-somatic compartment [78,79]. The afterhyperpolarization (AHP) that is observed after a burst of action potentials is due to the activation of voltage- and Ca^2+^-sensitive BK (fast component of AHP) and SK channels (medium and slow components of AHP) and contributes to the regulation of neuronal excitability. A reduction of the AHP is observed in hippocampal CA1 neurons from animals that learn hippocampal-dependent tasks such as trace eyeblink conditioning [80] or spatial water maze [81] and also following a long-term potentiation induced by a theta burst stimulation (TBS-induced LTP, see below) [82], suggesting the role of AHP regulation in learning and memory processes. Interestingly, in aged rodents presenting memory deficits, hippocampal neurons present a more robust AHP that induces decrement in basal excitability and an impaired ability to modulate excitability [83]. A similar phenomenon is observed in neurons overexpressing amyloid precursor protein (APP) [84] or neurons form the 5XFAD Alzheimer disease mouse model that express high levels of amyloid-β42 [85].

Hagenston and colleagues showed that glutamate acting on mGluR mediates an IP_3_-dependent wave of Ca^2+^ that activates SK channels responsible for AHP [86]. As TRPC channels are calcium-permeable, they could contribute to AHP. Interestingly, Neuner et al. developed a targeted proteomics approach and compared age-matched mice exhibiting a normal or an impaired contextual fear memory [59]. They showed that deficit in contextual fear memory was related to increased TRPC3 expression in the hippocampus. They also showed that, in normal mice, a successful contextual fear conditioning was associated to a reduction by one third in the expression of TRPC3. TRPC3 blockade or acute deletion reduced the AHP, increased excitability of hippocampal firing neurons, and enhanced contextual memory. Thus, the increased expression of TRPC3 observed in the hippocampus of mice exhibiting impaired contextual fear memory might actually be the cause of this cognitive deficit.

### 5.3. Persistent Activity

In some conditions, neurons are able to maintain a sustained firing after the synaptic stimulation or the depolarizing pulse is finished. This activity can persist for few seconds or minutes. It has been described in different brain regions involved in learning processes such as amygdala, entorhinal cortex, hippocampus, and prefrontal cortex (reviewed in [55,87]). In the hippocampus, a persistent activity contributes to working memory and associative learning [88,89]. Persistent activity is due to a burst-induced sustained depolarization (so called afterdepolarization) that reaches the firing threshold. This depolarization is itself due to an activity-dependent stimulation of Ca^2+^-activated non-selective cationic current (CAN). Experimentally, it can be triggered by a short depolarizing current in the presence of muscarinic or metabotropic glutamatergic agonists or by cholinergic afferent stimulation [22,90,91,92,93]. 

Synchronized burst firing of a large group of neurons can induce seizures. These seizures can be triggered e.g., by repetitive stimulations of the perforant pathway or by application of chemical convulsivants such as the muscarinic agonist pilocarpine [94] or the mGluR-specific agonist, (1S,3R)-1-aminocyclopentane-1,3-dicarboxylicacid (ACPD), or DHPG [95]. The lateral septum and the CA3 region of the hippocampus are two regions highly vulnerable to seizures and excitotoxicity (see below) where type I mGluR agonists induce important epileptiform burst firing with a large depolarizing plateau potential.

Both persistent firing and seizures seem triggered by the CAN current, the identity of which is not fully understood. Initially, El-Hassar and colleagues showed that stimulation of group I mGluR triggered IP_3_-dependent Ca^2+^ waves that contribute to an SK channel-mediated transient hyperpolarization (AHP, see above) followed by a sustained depolarization. On the basis of its sensitivity to blocking antibodies, this depolarization was attributed to the activation of TRPC1/4/5 channels [91]. Studying seizure mechanisms, Phelan and colleagues then demonstrated that *Trpc1^−/−^* and *Trpc1/4^−/−^* double knockout exhibited largely reduced depolarizing plateau potential and subsequent epileptiform bursts of activity induced by mGluR agonists in septal neurons and in CA1 pyramidal neurons [22,96]. However, pilocarpine-induced seizures were unaltered. In contrast, TRPC5 played a critical role in pilocarpine-induced seizures but not in mGluR-induced firing bursts [22,93]. This suggests that the presence of TRPC1 in the composition of the channel might be critical for its interaction with (and activation by) type I mGluR (see above). The involvement of TRPC channels in persistent activity might depend on the brain region and the neuronal circuit considered. Recently, using *Trpc1/4/5^−/−^* and hepta-knockout (*Trpc1/2/3/4/5/6/7^−/−^*) mouse lines, Egorov et al. demonstrated that TRPC channels were not required for graded persistent activity in neurons of the layer V of medial entorhinal cortex [97]. Similarly, Dasari et al. showed that TRPC5 and TRPC6 were not required for cholinergic excitation of pyramidal neurons of the medial prefrontal cortex [98]. However, using specific pharmacological blockers and antibodies, Arboit et al. confirmed the involvement of TRPC4 and TRPC5 in persistent firing of CA1 pyramidal cells [99]. Altogether, it seems that TRPC1 and -4 are responsible for the mGluR-induced depolarizing plateau potential, at least in the CA1 region, and their loss is associated to an impairment of working memory [16,57] (Figure 1).

## 6. Synaptic Plasticity 

Synaptic plasticity is the ability of synapses to change over time their strength and their efficacy of transmission in response to their pattern of activity [1,4,100]. This synaptic change can last few milliseconds to several minutes (short-term synaptic plasticity), hours, days, or even more (long-term plasticity). The various forms of short-term synaptic plasticity are thought to play important roles in short-term adaptations to sensory inputs, transient changes in behavioral states, and short-lasting forms of memory. They are triggered by specific short bursts of activity causing a transient accumulation of calcium in presynaptic nerve terminals, which in turn changes the probability of neurotransmitter release. Long-term potentiation (LTP) and its counterpart long-term depression (LTD) are the best described forms of long-lasting synaptic plasticity. It is widely assumed that LTP and LTD reflect synaptic and cellular processes that occur during formation/storage and extinction of memories, respectively. Various cellular cascades are triggered by different stimulation protocols used to induce LTP and LTD, all of which converge to produce changes in structure and function of dendritic spines at glutamatergic synapses, namely increased or decreased number of α-amino-3-hydroxy-5-methyl-4-isoxazolepropionic acid (AMPA) receptors and increased or decreased size of dendritic spines respectively. Both LTP and LTD typically depend on postsynaptic ionotropic glutamate N-methyl-D-aspartate receptor (NMDAR) [101] and Ca^2+^ for their induction and on protein synthesis for their maintenance. Other signaling pathways have, however, been involved, such as the activation of group I mGluRs and other PLC-coupled G-protein receptors that trigger the release of Ca^2+^ from internal stores. Blockade of group I mGluRs inhibits some forms of LTP and LTD [102] and disrupts hippocampus-dependent learning [103].

### 6.1. Short-Term Plasticity and Synaptic Transmission

Bröker-Lai et al. showed that in hippocampal neurons from *Trpc1/4/5^−/−^* mice, action potential-induced EPSCs were significantly reduced, whereas frequency, amplitude, and kinetics of quantal miniature postsynaptic were normal, demonstrating that TRPC1/C4/C5 complex facilitates synaptic transmission and possibly explaining the impairment of working memory observed in these mice (see above; [16]). The presynaptic role of these TRPC isoforms was confirmed by a recent study showing that loss of TRPC1/C4/C5 reduced the presynaptic rise of [Ca^2+^]_i_ induced by high frequency stimulation (HFS) and diminished the pool of readily releasable neurotransmitter vesicles and their replenishment rate during HFS [20]. Overexpression of TRPC1 or TRPC5 increased Ca^2+^ dynamics and replenishment rate in WT neurons but only the overexpression of TRPC5 could rescue the situation in *Trpc1/4/5^−/−^* neurons, possibly because in contrast to TRPC1, TRPC5 is able to form functional homomeric channels. Besides, neurons expressing TRPC5 presented a particularly strong short-term enhancement of synaptic response after 10 stimulation pulses. As TRPC5 are Ca^2+^-activated channels (in contrast to TRPC1, which are not [49]) functionally coupled to voltage-gated Ca^2+^ channels (VGCCs), the authors suggest that Ca^2+^ entry through VGCCs during action potential may trigger TRPC channel openings leading to additional Ca^2+^ influx and thereby modulating presynaptic Ca^2+^ dynamics and synaptic plasticity (Figure 1).

#### 6.1.1. LTP 

Experimentally, LTP can be induced by a HFS (typically 100 Hz for 1s) or by a more physiologically relevant stimulus, the TBS resembling to spike discharge patterns of hippocampal neurons in animals in exploratory situations and consisting in a series bursts of four or five pulses delivered at 100 Hz, repeated at 5 Hz (θ rhythm).

Expression of LTP is due to both pre- and postsynaptic mechanisms. For example, LTP is essentially presynaptic in the synapses between granule cells of the DG and the CA3 pyramidal neurons [104] and involves VGCCs that allow an increased influx of Ca^2+^ during AP (see above). In contrast, between the Schaffer collateral terminals and the CA1 pyramidal neurons, LTP essentially depends on postsynaptic modifications and is predominantly triggered by synaptic activation of NMDARs, upon strong post-synaptic depolarization and removal of the Mg^2+^ block from these receptors [101]. The required intracellular Ca^2+^ arising can nevertheless involve an influx through other channels such L-type Ca^2+^ channels, TRP channels or even from a release of Ca^2+^ from intracellular stores [105,106,107].

LTP has an early phase, which is essentially mediated by phosphorylation of existing proteins without synthesis of new proteins and a late phase that involves the activation of transcription factors and is dependent on protein synthesis. Many cellular mechanisms modulate LTP, such as stimulation of mGluR [108]. A chemical LTP can be induced by co-activation of NMDAR and mGluR in hippocampal CA1 neurons [109]. In addition, mGluR activation facilitates activity-dependent LTP in CA1 neurons [110], especially its late-phase LTP [111,112], and mGluR antagonists impair LTP [103]. Interestingly, it has been reported that activation of mGluR5 facilitates LTP generated by a weak tetanization paradigm such as TBS but fails to affect a robust LTP generated by strong tetanization [103].

We showed that TBS-induced LTP was reduced in *Trpc1^−/−^* mice, suggesting that TRPC1 exerts its effect in TBS-induced LTP following its activation by mGluR5 [57] (Figure 1). Accordingly, the residual LTP observed in brain slices from *Trpc1^−/−^* mice could not be further reduced by mGluR antagonists. In contrast, LTP induced by a strong tetanization (HFS) of Schaffer collaterals was reported to be normal in *Trpc1^−/−^*, *Trpc1/4^−/−^*, and in *Trpc1/4/5^−/−^* [57,93]. Genetic ablation of TRPC7 was also shown to disrupt HFS-induced LTP at CA3-recurrent collateral synapses and at Schaffer collaterals-CA1 synapses where it could contribute to the initiation of epileptiform bursts and seizures respectively [26]. 

The situation for TRPC5 is less clear. HFS-induced LTP is reduced in *Trpc5^−/−^* but not in *Trpc1/4/5^−/−^* at Schaffer collaterals-CA1 synapses [16,93], but the reason of the apparent discrepancy is unclear.

Interestingly, among the TRP channels from subfamilies other that TRPC, TRPM4 has been demonstrated to play an important role in LTP of Schaffer collaterals-CA1 synapses. Being Ca^2+^-sensitive but Ca^2+^-impermeable, TRPM4 creates a feed-forward loop that generates the post-synaptic membrane depolarization that is necessary to fully activate NMDAR during the induction of LTP [113].

#### 6.1.2. LTD

Like LTP, LTD is predominantly mediated by the activation of NMDAR (NMDAR-LTD) or by the activation of mGluRs (mGluR-LTD). Experimentally, NMDAR-LTD can be induced by a low-frequency stimulation (1 Hz, 15 min), whereas mGluR-LTD can be obtained via paired-pulse low frequency stimulation or pharmacologically with the group I mGluR agonist DHPG [114]. LTD can reverse a beforehand reinforced synapse and corresponds to a “depotentiation” (i.e., a kind of erasure of synaptic memory trace) or weakens and/or eliminates naive synapses, a requirement to refine and consolidate memories [115]. mGluR-LTD thus plays a key role in reversal learning and in cognitive flexibility, allowing a suppression of previously acquired memory and acquisition of new information to adapt to novel situations [116,117,118]. We observed that mGluR-LTD is impaired in brain slices of *Trpc1^−/−^* mice [58]. This correlates with an alteration of reversal spatial learning. The mechanism is not fully understood but involves a decrease of extracellular signal-regulated kinases 1/2 (ERK1/2) activation with a subsequent decrease of expression of the activity-regulated cytoskeletal-associated protein Arc, an immediate early gene that plays a key role in AMPA receptors endocytosis (Figure 1).

The involvement of other TRPC isoforms has not been studied so far in this form of plasticity in the hippocampus, but TRPC3 activation seems to be involved for the induction of LTD in cerebellar Purkinje cells [119].

## 7. Adult Neurogenesis

Except for the TRPC1 isoform, few studies have been devoted to the role of TRPCs in neurogenesis. However, as TRPCs are involved in the control of cell proliferation and migration, they could play an important role in neurogenesis, which seems to be increasingly involved in several types of memory and whose progressive decrease with age or disease could be implicated in memory loss.

### 7.1. Neurogenesis and Memory

In fact, it has long been considered as a dogma that adult brain has no capacity for generating new neurons. However, for a few decades now, the use of different techniques such as incorporation of bromodeoxyuridine or cell-type specific immunostaining has clearly demonstrated that adult neurogenesis originating from neural progenitor cells occurs in two regions of the brain, in the subventricular zone (SVZ) of the lateral ventricle and in the subgranular zone (SGZ) of the DG (reviewed in [120]). The process was first observed in rodent animal models, but it seems also to occur in other mammals, including humans [121,122,123]. In the two discrete germinal niches, adult neural stem cells (aNSC) proliferate and self-renew. After asymmetric division, they can also generate either glial cells, such as astrocytes or transient amplifying progenitors, giving rise to neuroblasts that are able to proliferate and to differentiate into neurons. Neurons originating from the subventricular zone migrate to the olfactory bulb whereas neurons born in the SGZ of the DG integrate into the local neural network of the DG. In the mouse DG, newly born neurons can account for up to ten percent of the granule cell population [3,124]. It is much weaker in humans, but, nevertheless, even if generation of new neurons is slow, it can modify the neuronal network and therefore add a level of possible regulation to the brain plasticity described so far.

Despite some controversies, it seems that adult-born neurons are not necessary for the initial acquisition of most hippocampus-dependent memories, such as spatial learning (Morris water maze) or contextual fear conditioning [125,126]. Continuous addition of new neurons in the DG can nevertheless be expected to disturb its pre-existing structure and previously established connections [127,128]. It could therefore theoretically be involved in memory clearance or depotentiation (see above). Accordingly, experiments show that physically or genetically impaired neurogenesis is accompanied by a facilitated long-lasting maintenance of LTP measured in vivo in the DG [129].

A correlated function in which adult neurogenesis seems to play an important role is memory consolidation [130]. Hippocampus is crucial for the recall of recent memory but not for the recall of remote memory, which occurs within a distributed cortical network. Kitamura and colleagues demonstrated that decreased neurogenesis prolongs the hippocampus-dependent period of associative fear memory and inhibits cortical memory consolidation [129].

Another function of adult neurogenesis seems to be pattern separation, i.e., in the ability to discriminate between two pieces of similar contextual or spatial information [131]. DG receives projections from the entorhinal cortex. However, the number of granule cells is much larger that the number of entorhinal cells projecting onto the DG [132]. Cortical inputs can disperse over this large number of granule cells that are sparsely activated but that have strong synapses onto CA3 pyramidal cells. The DG thus concentrates the information and allows the transformation of overlapping patterns of activity coming from the cortex into less overlapping patterns of activity in the CA3 region.

### 7.2. SOCE and Neurogenesis

At each state of differentiation, aNSC and their daughter cells express different specific markers and are influenced by extrinsic and intrinsic factors such as morphogens, growth factors, neurotransmitters, cytokines, adhesion molecules, and transcription factors (reviewed in [126]). Transcriptome studies underlined elevated expression levels of a specific subset of genes related to Ca^2+^ signaling in aNSC [133].

Ca^2+^ signaling has been described to regulate stem cell quiescence and proliferation in many cell types [134,135,136] including in several cancer cell types [137]. Ca^2+^ influx through multiple pathways ha been implicated but SOCE is probably the most relevant pathway in proliferation of aNSC as it allows sustained Ca^2+^ influxes in response to a wide range of environmental factors [138]. aNSC from the SVZ and from the SGZ express molecular actors of SOCE, including TRPC1, Orai1, and STIM1 [139,140], and TRPC1 is the most upregulated TRPC isoform under proliferative conditions. Recent studies showed that pharmacological inhibition of SOCE or genetic silencing of TRPC1, Orai1, or STIM1 decreases the amplitude of SOCE and inhibits proliferation of aNSC, causing a cell cycle arrest at the G0/G1 phase [140]. Moreover, SOCE inhibition controls aNSC self-renewal by diminishing symmetric (proliferative) for the benefit of asymmetric division [139].

### 7.3. Factors Influencing Neurogenesis

Hippocampal neurogenesis is presumed to persist throughout the lifespan; however, a decline in neurogenesis occurs with age [141]. This might be due to a progressive deprivation of important regulators of neurogenesis such as BDNF and CREB [142]. Adult hippocampal neurogenesis is also influenced by many pathological stimuli such as ischaemia, seizures, diabetes, inflammation, and neurodegenerative diseases such as Alzheimer disease (AD) [143]. Interestingly, two recent studies show that adult hippocampal neurogenesis is impaired at early stages of AD suggesting that the process might promote AD-related cognitive deficits [144,145]. Obviously, stimulating neurogenesis could therefore serve as a therapeutic target for improving cognitive function [146].

In rodent animal models, it has been clearly shown that aerobic exercise enhances neurogenesis, learning, and long-term potentiation in mice [147]. Currently, exercise is therefore considered as a serious preventive approach to diminish cognitive decline related to Alzheimer disease, even if the mechanisms of this beneficial action are not fully understood [148]. Another situation that can improve adult neurogenesis is related to environmental enrichment (EE). In humans, EE involves a combination of increased social interaction, physical exercise, and continuous exposure to learning tasks. It was shown to protect against AD and other neurodegenerative diseases [149]. Experimentally with rodent animals, EE refers to housing conditions in which animals are exposed to diverse objects in a huge living space. EE improves learning and memory abilities, enhances hippocampal neurogenesis, and promotes LTP [150,151,152]. Enhanced hippocampal functions result at least partially from activity-dependent increases in the levels of mGluR5, Homer1c, and phospho-p70S6 kinase [153]. Recently, Du et al. [154] observed that TRPC1 knockout abolished the EE-induced spatial memory enhancement and EE-induced LTP and neurogenesis in the DG, whereas exogenous expression of TRPC1 could rescue the EE-associated cognitive functions, LTP induction and hippocampal neurogenesis. EE could stimulate the expression of TRPC1, which, in turn, could regulate the activity of ERK/p38/CREB pathway. TRPC1 might modulate the expression of several proteins that could play a role in memory, such as α-internexin and glia maturation factor β [56].

## 8. Cell-Death Excitotoxicity

TRPC channels also seem involved in neuronal apoptosis or survival. Bollimuntha and colleagues showed that TRPC1 protected human SH-SY5Y cells against apoptosis induced by different neurotoxins [155]. This might explain the observation that memory deficits and apoptosis induced by amyloid-β are exacerbated in *Trpc1^−/−^* mice [156]. Similarly, TRPC1 was shown to inhibit apoptosis of neuronal cells in basal ganglia [157]. 

In contrast, TRPC channels might be involved in excitotoxicity, a type of neuronal death due to an excessive stimulation by neurotransmitters such as glutamate. Excitotoxicity is triggered by an excessive influx of Ca^2+^. It involves the NMDAR, which is highly permeable to Ca^2+^ [158,159], but also the mGluR [160]. Several studies report that glutamate activates cell death by massive increases in [Ca^2+^]_i_ via the TRPC1 channel [161,162]. As explained above, TRPC1, TRPC4, and TRPC5 are also involved in pilocarpine-induced seizures and in mGluR-induced epileptiform firing bursts, as well as in subsequent excitotoxicity [22,93,96].

## 9. Conclusions and Perspectives

In the hippocampus, TRPC channels are clearly involved in the control of cell excitability, in synaptic plasticity, and in cell survival and neurogenesis. They thus modulate different forms of memory and learning processes. It is therefore tempting to hope that modifications of expression or activity of these channels could ameliorate memory performances. So far, no TRPCs have been causally linked to a neurological disease, but their involvement in neuropsychiatric and neurodegenerative disorders yet remains to be fully explored (reviewed in [163,164,165]). However, the large distribution of the TRPC channels in the brain and in almost all organs and their involvement in so many fundamental cellular functions preclude, at least at the present time, the use of TRPC modulators as therapeutic agents. Nevertheless, the use of molecules specifically activating or inhibiting certain TRPC isoforms can be rich in information. For example, Cheung et al. showed that (-)-englerin A, a potent activator of TRPC4 and TRPC5 used as an anti-cancer cytotoxic agent, produced adverse effects of major anxiety [166]. These effects were not observed in TRPC4/TRPC5 double knockout mice. Just et al. reported that HC070, a TRPC4/5 inhibitor attenuated neuronal activity recorded from the amygdala and had anxiolytic and antidepressant effects in mice, suggesting the use of this molecule to treat generalized anxiety disorder [167]. Previously, another TRPC4/C5 inhibitor, the benzimidazole-based compound M084, was reported to have inhibitory effect in the range of μM on TRPC1/4/5 channels and anxiolytic and antidepressant effects in mice by acting on BDNF and its downstream signaling [168]. Nowadays, one of the most specific and promising TRPC1/C4/C5 channel blockers is Pico145. This molecule, originally disclosed in a patent published by Hydra Biosciences [169], revealed a potency ranging from pM to nM on the different TRPC1/4/5 subtypes [170]. We treated hippocampal slices with Pico145, showing that the acute inhibition of TRPC1/C4/C5 channel impaired both TBS-induced LTP and mGluR-LTD, similarly to the genetic depletion of *Trpc1* gene [57,58]. Moreover, the in vivo administration of Pico145 in mice caused an impairment of memory extinction, suggesting a potential use of Pico145 for the treatment of pathologies in which the process seems exaggerated, such as in Fragile X syndrome and autism [171,172].
